# The Peptide Oxytocin Antagonist F‐792, When Given Systemically, Does Not Act Centrally in Lactating Rats

**DOI:** 10.1111/jne.12331

**Published:** 2016-04-25

**Authors:** G. Leng, J. A. Russell

**Affiliations:** ^1^ Centre for Integrative PhysiologyUniversity of EdinburghEdinburghUK

**Keywords:** electrophysiology, milk‐ejection reflex, lactation, oxytocin, paraventricular nucleus

## Abstract

Oxytocin secreted by nerve terminals in the posterior pituitary has important actions for ensuring a successful outcome of pregnancy: it stimulates uterine contractions that lead to birth and it is essential in the milk‐ejection reflex, enabling milk to be expelled from the mammary glands into the mouths of suckling young. Oxytocin also has important actions in the brain: released from dendrites of neurones that innervate the posterior pituitary, oxytocin auto‐excites the neurones to fire action potentials in co‐ordinated bursts, causing secretion of pulses of oxytocin. Central oxytocin actions are blocked by an oxytocin antagonist given into the brain and, consequently, milk transfer stops. Systemic peptide oxytocin antagonist (atosiban) treatment is used clinically in management of pre‐term labour, a major obstetric problem. Hence, it is important to know whether an oxytocin antagonist given peripherally can enter the brain and interfere with central oxytocin actions. In the present study, we tested F792, a peptide oxytocin antagonist. In urethane‐anaesthetised suckled rats, we show that the mammary gland responsiveness to oxytocin is blocked by i.v. injections of 7 μg/kg of F792, and the milk‐ejection reflex is blocked when F792 is given directly into the brain at a dose of 0.2 μg. To critically test whether F792 given systemically can enter the brain, we recorded the suckling‐ and oxytocin‐induced burst‐firing of individual antidromically identified oxytocin neurones in the paraventricular nucleus. Given systemically at 100 μg/kg i.v., F792 acted only peripherally, blocking the milk‐ejecting actions of oxytocin, but not the burst‐firing of oxytocin neurones during suckling (n = 5 neurones in five rats). Hence, this peptide oxytocin antagonist does not enter the brain from the circulation to interfere with an essential oxytocin function in the brain. Furthermore, the functions of oxytocin in the brain evidently cannot be explored with a systemic peptide antagonist.

The essential role of oxytocin in mammals is in eliciting the transfer of milk from the alveoli in the mammary glands into the mouths of the suckled young. The intermittent secretion of large pulses of oxytocin from the posterior pituitary gland during suckling, or as a conditioned reflex response to imminent suckling, forms the efferent limb of the milk‐ejection reflex 1. Without oxytocin, as in oxytocin‐deficient mice, milk cannot be transferred and the young perish 2, 3.

In their landmark studies of the mechanisms of the milk‐ejection reflex, Wakerley and Lincoln 4, 5, 6 showed that, in the lactating rat, oxytocin is secreted in pulses a few minutes apart during suckling, with each pulse being the result of a high‐frequency burst of action potentials in the magnocellular oxytocin neurones, and Summerlee and Lincoln 7 showed that the same behaviour occurs in conscious rats as in anaesthetised rats. Importantly, Wakerley and Lincoln 4 showed that the bursts were not a response to any actions of circulating oxytocin, injected in amounts equivalent to the sharp increases that underlie each milk ejection. Hence, the electrical and secretory activity of the oxytocin neurones was not influenced directly by circulating oxytocin. However, to excite the release of sufficient oxytocin to elicit a milk ejection, many if not all magnocellular oxytocin neurones must fire a burst of action potentials within a narrow time window, achieving near synchrony. Oxytocin neurones in the four magnocellular nuclei indeed show such co‐ordinated burst‐firing during suckling 8, oxytocin is released in the nuclei in response to suckling 9 and centrally administered oxytocin will dramatically facilitate the milk‐ejection reflex 10, acting both in the magnocellular nuclei 11 and perhaps at other central sites 12, 13, 14. Critically, small amounts of an oxytocin antagonist, given i.c.v. or directly into just one supraoptic nucleus, completely block the milk‐ejection reflex 11; thus, the source of this oxytocin is likely to be the dendrites of the magnocellular oxytocin neurones themselves.

The mechanisms by which such dendritic oxytocin release is stimulated and then acts to co‐ordinate burst‐firing is complex, involving local inhibitory and excitatory interactions in the dendritic microenvironment 15. These interactions are posited to loosely couple the magnocellular oxytocin neurones to enable and trigger co‐ordinated burst‐firing. The data and inferences from many studies have been used to develop a computational model that replicates the electrophysiological behaviour of magnocellular oxytocin neurones during suckling; hence, the assumptions used in this model about how the identified variables interact are validated 16.

Oxytocin also has an important role in supporting the birth of young through its powerful uterotonic actions, even if this role does not appear to be absolutely essential, unlike its importance in lactation 17. Oxytocin is considered to be more important normally in further driving established parturition than in its initiation 18, 19, although oxytocin can advance the onset of birth in rats 20 and oxytocin antagonists can delay it 21. Because threatened pre‐term labour, a major health issue worldwide, may be a result of the premature secretion of oxytocin, antagonists of oxytocin action at the oxytocin receptor have been developed for possible use in the prevention of progression of pre‐term labour to pre‐term birth. Presently, atosiban, a peptide antagonist, is in clinical use with some success 22, 23, 24, and nonpeptide antagonists have also been developed 25, 26. In view of the central actions of oxytocin considered here in parturition and lactation, it is important that these actions in the brain are not compromised near the end of pregnancy by drugs aimed at preventing pre‐term birth.

Magnocellular oxytocin neurones also fire in intermittent, intense bursts in association with the delivery of young 27, and oxytocin is released within the magnocellular nuclei during birth 28. Importantly, if the action of oxytocin on magnocellular neurones is blocked by infusion of an oxytocin antagonist into the supraoptic nucleus, the local release of oxytocin is reduced and parturition is prolonged 28, and so the local release of oxytocin is likely to promote the burst‐firing of oxytocin neurones during birth as it does during suckling.

Oxytocin is released into the brain not only from the axons of parvocellular neurones, the cell bodies of which are located in the paraventricular nuclei 29, but also from dendrites of magnocellular neurones 30, 31 and, as shown recently, from centrally projecting axons of these neurones 32. Such centrally released oxytocin has central actions that are important for the transition from pregnancy to motherhood, underpinning the optimal patterning of oxytocin secretion for birth and milk transfer, and including the facilitation of maternal behaviour 3, 33.

In the present study, we examined whether peripheral administration of a peptide oxytocin antagonist (F792, 34,35) can have actions in the brain. Using the urethane‐anaesthetised suckled milk‐ejecting rat model, we tested the hypothesis that this antagonist will block the milk‐ejection reflex when given directly into the brain, whereas, if given systemically, it will only act peripherally, blocking the milk‐ejecting actions of oxytocin but not the burst firing of oxytocin neurones that normally occurs during suckling.

## Materials and Methods

### Animals

Female Sprague–Dawley rats, weighing 200–250 g and obtained from Bantin & Kingman (Hull, UK) were housed under a 12 : 12 h light/dark cycle (lights on 07.00 h) at 20–22 ^°^C with free access to food and water. Mating was carried out in‐house, as required, to generate lactating rats. All procedures were performed in accordance with UK Home Office regulations governing animal experimentation with institutional ethical approval. Rats were initially housed six per cage but were housed individually once mating was confirmed by finding a vaginal plug beneath the grid floor of the mating cage.

### Intramammary pressure recording

After birth, the rats were kept with their litters (range 8–14 pups, mean ± SEM, 11.5 ± 0.4, n = 13) until days 5–13 of lactation, when all but one to three pups were removed overnight and kept in a cage with abundant bedding, prior to the experiment the next morning. Overnight separation ensures rapid attachment to the nipples, as well as ample milk availability. For the experiment, the dams were anaesthetised with urethane (intraperitoneal ethyl carbamate, 1.1 g/kg, 25% w/v solution; Sigma, St Louis, MO, USA) and the main milk duct of two mammary glands was cannulated via the nipple to record intramammary pressure continuously via pressure transducers (calibrated with a water manometer at 100 and 200 mm water) coupled to a CED 1401 interface and a PC running spike, version 2 (Cambridge Electronic Design, Cambridge, UK). A jugular vein was cannulated for injection of oxytocin (10 mU/ml 0.9% saline; Sigma) or F792 (up to 100 μg/ml 0.9% saline; a peptide oxytocin antagonist: dCys‐d‐Trp‐Ile‐allolle‐Asn‐Carba‐6‐NmeOrn‐NH_2,_ kindly provided by Per Melin (Ferring Institute, Malmö, Sweden) 34, 35. Intermittent bolus i.v. injections of oxytocin (from 0.5–5 mU) were given to assess mammary gland responsiveness, and to compare with responses to suckling‐induced milk ejections. Mammary gland sensitivity to i.v. oxytocin injection was established initially by injecting oxytocin in doses from 0.1–2 mU, given > 10 min apart.

#### Cannulation

A cannula was implanted stereotaxically in all rats for injection of oxytocin or F792 into a lateral cerebral ventricle. With the rat's head in a stereotaxic frame, a hole was drilled into the skull, 1.6 mm lateral and 0.6 mm caudal to bregma, and a 22‐gauge i.c.v. guide cannula (Plastics One; Roanoke, VA, USA) was lowered with its tip 3.5 mm below the skull surface, and fixed in place with dental acrylic and two small screws secured in the skull. Injections [of oxytocin, 1 mU/μl artificial cerebrospinal fluid (aCSF) or F792, 0.05 μg/μl] were given via a 28‐gauge cannula, with its tip 1 mm below the guide cannula tip, connected via polythene tubing to a Hamilton microsyringe.

#### Suckling‐induced oxytocin secretion

The milk‐ejection reflex can be observed under urethane anaesthesia 4, 5, 6. Typically, if hungry pups are applied to the nipples 2 h or more after any surgical interference with the rat, then, after a latent period of 20–40 min, oxytocin cells begin to display occasional high‐frequency bursts of spikes. These bursts are synchronised amongst the oxytocin cells and so give rise to the secretion of pulses of oxytocin into the systemic circulation. If sufficiently large, these result in abrupt increases in intramammary pressure of 5–10 mmHg that occur approximately 12 s after the recorded bursts and are similar to those produced by bolus injections of 0.5–1 mU. The frequency and amplitude of the recorded bursts vary considerably between rats and between cells within rats, and their magnitude also depends upon the number of pups suckling and the interval between bursts varies over the course of an experiment. However, for any given cell, the magnitude and profile of milk‐ejection bursts varies little from one burst to the next. Not all rats will show an intact reflex but, in many that do not, the reflex can be initiated by i.c.v. injection of 2 mU of oxytocin. In these experiments, the suckling stimulus was provided by a litter of eight to 10 pups applied to the uncannulated nipples, with at least six pups attached throughout.

### Electrophysiological recording

Extracellular electrophysiological recordings of the spiking activity of magnocellular oxytocin neurones were made from antidromically‐identified cells within the paraventricular nucleus via a dorsal approach 4, 5. With the urethane‐anaesthetised rat's head held in a stereotaxic frame, and the skull levelled between bregma and lambda, a rectangular hole (approximately 25 mm^2^) was made with a burr through the skull (rostrally from 0.5 mm caudal, laterally from 1.8 mm lateral to bregma) to allow a glass recording electrode (filled with 0.9% NaCl, tip of approximately 1 μm) to be lowered into the paraventricular nucleus 7.5 mm below the dorsal surface of the brain. A concentric bipolar stimulating electrode (SNEX‐100; Clark Electromedical Instruments, Reading, UK) was lowered on to the pituitary stalk through another hole, made on the midline 1.5 mm rostral to lambda at an angle calculated to intersect with the pituitary stalk at a depth of 9 mm from the surface of the brain. Correct positioning of the stimulating electrode tip was assessed by applying biphasic pulses (0.1–10 mA peak‐to‐peak at 50 Hz for 2 s) and noting an accompanying increase in intramammary pressure produced as a result of evoked oxytocin secretion from the posterior pituitary gland.

Magnocellular neurones were identified from the constant latency of antidromic spikes generated by pituitary stalk stimulation. Oxytocin neurones were distinguished from vasopressin neurones by their intermittent high‐frequency burst discharge during suckling. Discriminated spikes were recorded using a CED 1401 interface connected to a PC running spike, version 2 36. Milk‐ejection bursts were recognised as a sequence of > 20 spikes occurring with unusually short interspike intervals; the first interval of less than 40 ms in a sequence was taken as the start of each burst for purposes of quantification of burst amplitude. The illustrated profiles show instantaneous frequencies: these plot a point at the time of each spike occurrence at a value proportional to the inverse of the preceding interspike interval (see Results).

### Experimental design and analysis

Experiment 1 was designed to test antagonism by F792 of milk‐ejecting actions of exogenous oxytocin, and to determine the dose for maximum effect. Increasing doses of oxytocin were given as bolus i.v. injections; doses were 0.5, 1, 1.5 and 2 mU (1 mU = 2.2 ng), which covers the range secreted per milk ejection during suckling. On the basis that 1 mU of oxytocin generally elicits a near maximal peak intramammary pressure response, this dose was initially used to test antagonism by F792, given in increasing i.v. doses from 0.02 μg, at approximately 10‐min intervals until effect, which was taken as complete prolonged suppression of oxytocin effects. Duration of effect was monitored for up to 2 h in three lactating rats.

Experiment 2 was designed to demonstrate blockade by F792 given by i.c.v. injection of suckling‐induced milk ejections triggered by i.c.v. oxytocin. Mammary gland sensitivity to 1 mU of oxytocin i.v. was assessed; pups were put to the nipples and, when at least six were suckling for several minutes, 2 mU of oxytocin was infused via the i.c.v. cannula over 30 s. Following a series of milk ejections, 0.1 μg of F792 was infused via the i.c.v. cannula; if milk ejections continued, further i.c.v. F792 injections were given to achieve the maximum effect of stopping milk ejections. Mammary gland sensitivity to oxytocin was regularly tested over the following 2 h, and i.c.v. injections of oxytocin (up to 5 mU) were given to see whether the effects of the antagonist declined. Five suckled lactating rats were studied.

Experiment 3 tested whether systemically administered F792 inhibits burst‐firing of magnocellular oxytocin neurones in the paraventricular nucleus induced by suckling and triggered by i.c.v. oxytocin administration. For this experiment, extracellular recording of an antidromically identified magnocellular oxytocin neurone was established, and pups were applied to the nipples, as in Experiment 2. To facilitate suckling‐induced burst‐firing, oxytocin (1 mU) was infused via the i.c.v. cannula. Once milk‐ejection bursts of firing were triggered, 100 μg/kg of F792 was injected i.v. (this dose is > 100‐fold greater than the i.c.v. dose that blocked the milk‐ejection reflex). Five lactating rats were used in this experiment, and milk‐ejection bursts before and after i.c.v. F792 were compared within rats.

## Results

### Experiment 1: Antagonism by F792 of i.v. oxytocin action on intramammary pressure

**Figure 1:**
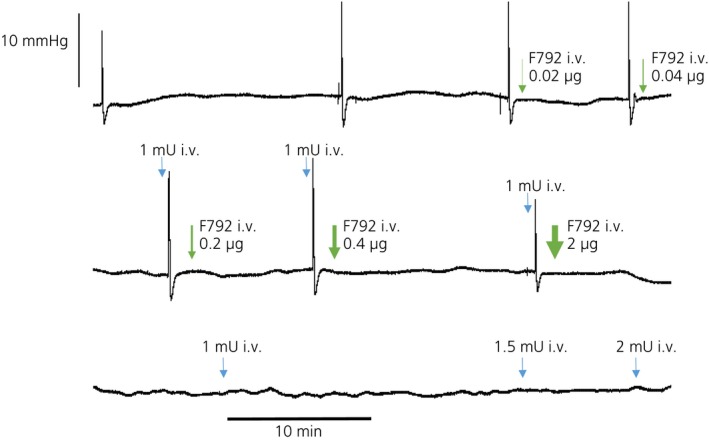
Determination of the i.v. dose of F792 (OTA) that blocks the milk‐ejecting action of oxytocin (OT). Intramammary pressure recording in urethane‐anaesthetised lactating rats (day 9); rats were not suckled. Bolus i.v. doses of 0.5, 1.0, 1.5, 2 mU of oxytocin (1 mU = 2.2 ng) were given at approximately 10‐min intervals. F792 was given after bolus injections of 1 mU of oxytocin in increasing doses (as shown) until there was no detectable response to subsequent oxytocin injections. Oxytocin action was blocked for > 80 min after injection of the 2‐μg dose (cumulative 2.66 μg) of F792.

Three nonsuckled lactating rats were used to determine a maximally effective peripheral dose of F792. A cumulative dose of 2.66 μg of F792 (7.23 μg/kg body weight) abolished any increase in intramammary pressure in response to bolus injections of 1–2.5 mU of oxytocin (i.v., given every 15–20 min) in all three rats for at least 90 min after the last injection of F792 (Fig. 1).

### Experiment 2: Interruption of reflex milk‐ejections by central F792 administration

**Figure 2:**
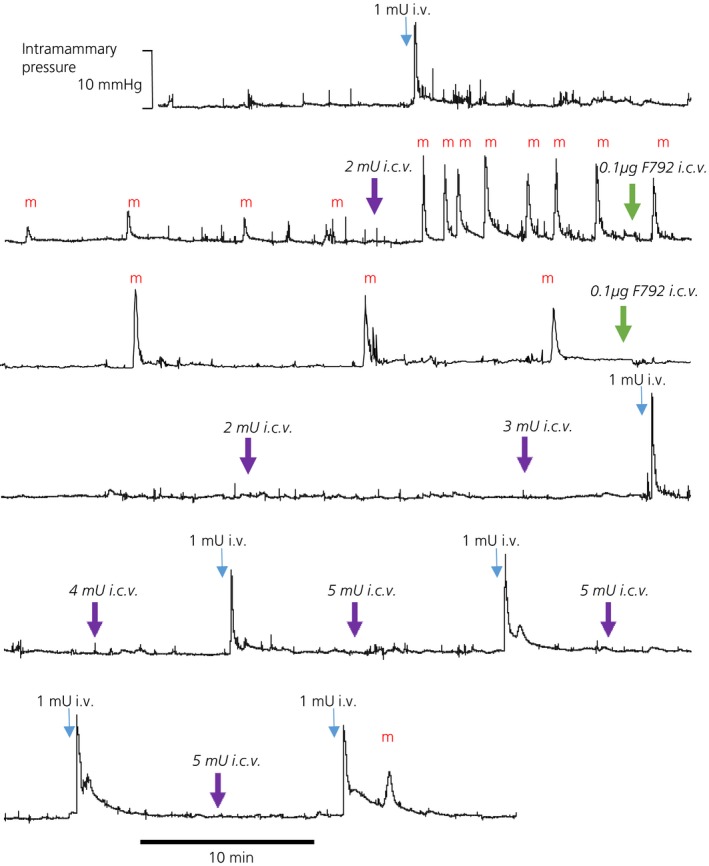
Intramammary pressure recording in a urethane‐ anaesthetised lactating rat (day 11/12, litter of 10 pups) at least six pups suckling throughout: the trace is a continuous recording. A bolus i.v. dose of 1 mU oxytocin demonstrated mammary gland sensitivity to oxytocin, and, in response to the suckling stimulus, reflex milk ejections (m) were observed. Oxytocin (2 mU) given i.c.v. triggered a sequence of 8 large milk ejections a few minutes apart. After an i.c.v. infusion of 0.1 μg F792 three further milk ejections were seen, but after a second i.c.v. infusion of 0.1 μg F792 no further spontaneous milk ejections were observed, even after i.c.v. infusions of 2, 3, 4, and 5 mU oxytocin. Bolus i.v. doses of 1 mU oxytocin elicited sharp increases in intramammary pressure similar to the initial response, showing intact sensitivity of the mammary gland to oxytocin. About 90 min after the second injection of F792 one small spontaneous milk ejection (m) was observed.

Milk ejections were considered to be established when three or more successive sharp increases in intramammary pressure were recorded (Fig. 2) either as a result of just the pups suckling or in combination with i.c.v. oxytocin administration. Then, F‐792 was administered i.c.v. in experiments on five suckled rats. Three of these rats exhibited spontaneous milk‐ejections and two required 2 mU and 3 mU of i.c.v. oxytocin, respectively, before suckling‐induced increases in intramammary pressure were recorded. In these experiments, 0.1–3 μg of i.c.v. F792 abolished both spontaneous and i.c.v. oxytocin facilitated milk ejections (Fig. 2), beginning with the highest dose in the first experiment and progressively lower doses in subsequent experiments. Sequential doses of 2, 3, 4 and then 5 mU of i.c.v. oxytocin (1 mU/μl, aCSF) every 15 min were ineffective at re‐establishing reflex milk‐ejection activity for at least 90 min. In all five rats, 1 mU of i.v. oxytocin remained capable of producing an undiminished increase in intramammary pressure throughout the experiments (Fig. 2).

### Experiment 3: F792 given i.v. and burst‐firing of oxytocin neurones during suckling

**Figure 3:**
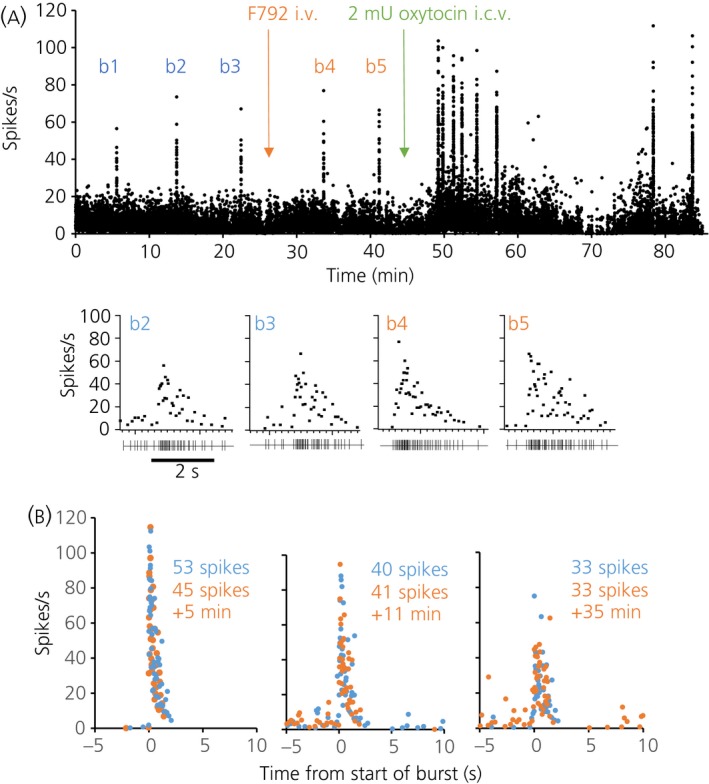
Demonstration that systemically administered F792 does not interfere with the excitation of magnocellular paraventricular nucleus oxytocin neurones during suckling. (a) Instantaneous firing rate record of a single antidromically identified paraventricular nucleus oxytocin neurone in a urethane‐anaesthetised lactating rat with at least six pups suckling throughout. b1, b2 and b3 indicate three spontaneous milk‐ejection bursts, after which 100 μg of F792 was injected i.v., as shown by the orange arrow. b4 and b5 mark two subsequent milk‐ejection bursts, after which 2 mU of oxytocin was injected i.c.v., producing the expected facilitation of burst amplitude and frequency. The profiles of the two bursts before injection of F792 and the two bursts after are expanded below. (b) Comparison of milk‐ejection burst profiles before and after i.v. injection of 100 μg of F792. Blue dots are from the last spontaneous milk ejection before injection of F792, and the orange dots are from the first burst after that injection. The total number of spikes within 2 s from the start of the burst is indicated, as is the time elapsed after injection of F792. The three cells (in three different rats) displayed bursts of differing magnitudes and amplitudes but, in each case, the burst amplitude and profile was completely unaffected by F792 injection.

In each of five experiments, an antidromically‐identified oxytocin neurone displaying suckling‐induced milk‐ejection bursts was recorded before and after i.v. injection of 100 μg of F792. In all five experiments, i.v. F792 had no effect on the bursting activity of these neurones, despite producing a complete and long lasting block of mammary gland responses in response to i.v. oxytocin (measured in three of the five experiments). In one of these experiments, i.c.v. injection of 2 mU of oxytocin before administration of the antagonist evoked five bursts containing 78 ± 3 spikes (mean ± SEM) within 2.5 s. At 25 min after injection of F792, a second i.c.v. injection of 2 mU of oxytocin evoked four bursts containing 79 ± 3 spikes within 2.5 s; no other bursts were observed in this experiment. In a second experiment (Fig. 3), three bursts were observed before administering F792, with 28, 32 and 32 spikes, respectively, within 2 s. After i.v. injection of F792, two further bursts were observed with 41 and 43 spikes within 2 s. Injection of 2 mU of oxytocin i.c.v. produced the expected facilitation of burst amplitude and frequency, eliciting five bursts with between 74 and 84 spikes within 2 s (mean 78 ± 1 spikes), followed by two later bursts (76 and 77 spikes within 2 s). In the other three experiments, as in the first two experiments, bursts that followed i.v. injection of F792 were indistinguishable from those observed before injection. Figure 3 shows the profile, for each of these experiments, of the last burst observed before injection of F792, with the first burst observed after.

## Discussion

The first experiment reported in the present study provided evidence for the effectiveness of F792 as a peptide antagonist of oxytocin action with respect to its essential role in effecting ejection of milk in lactation. A cumulative i.v. dose of 7.2 μg/kg body weight was effective against bolus i.v. injections of oxytocin in the physiological range [i.e. as secreted during sucking (4–6) for at least 90 min]. The second experiment tested whether any entry of F792 into the brain would interfere with the milk‐ejection reflex in anaesthetised lactating rats. This showed that i.c.v. injection of the antagonist abolished suckling‐induced milk ejections, as registered by cessation of sharp brief increases in intramammary pressure, whether or not i.c.v. injection of oxytocin was given to activate the milk‐ejection reflex. The lowest dose of i.c.v. F792 given (0.1 μg) inhibited milk ejections for 90 min from injection, and this inhibition was not overcome by repeated i.c.v. oxytocin injections, even at cumulative oxytocin doses six‐fold greater than were effective in evoking milk ejections before the i.c.v. F792 infusion. There was an eventual return during continuous suckling of intermittent increases in intramammary pressure, although of much reduced amplitude, without a reduction in mammary gland responses to bolus i.v. oxytocin injections. This resumption of milk ejections indicates reversibility, which may reflect clearance of F792 from the brain. Hence, we infer that, given i.c.v., F792 antagonises the actions resulting from oxytocin released in the brain that are essential for the milk‐ejection reflex. From previous studies, it is likely that, similar to other oxytocin antagonists, F792 blocks the priming and auto‐excitation of burst‐firing of oxytocin neurones during suckling that is dependent on the somato‐dendritic release of oxytocin by the magnocellular oxytocin neurones themselves 11, 16. Further evidence for the importance of local oxytocin in auto‐excitation during suckling is that local inhibition of leucine aminopeptidase, which is released by oxytocin neurones and inactivates extracellular oxytocin, has effects similar to i.c.v. oxytocin with respect to promoting burst‐firing 37.

On this background, the third experiment was designed to critically test whether F792 could enter the brain from the circulation when present at peripheral levels supramaximal for blocking oxytocin actions on the mammary gland. We found that, in this state, suckling‐stimulated burst‐firing of magnocellular oxytocin neurones in the paraventricular nucleus continued undiminished, with the complete absence of mammary gland milk‐ejection responses after each burst and after i.v. injection of test doses of oxytocin. Moreover, i.c.v. injections of oxytocin still evoked increased background firing activity and burst‐firing of the oxytocin neurones (Fig. 3), indicating intact auto‐excitatory oxytocin actions in the face of highly effective peripheral F792 levels.

Hence, we conclude that the peptide oxytocin antagonist F792 does not enter the brain to interfere with the actions of oxytocin released in the brain, at peripheral concentrations that strongly suppress peripheral oxytocin actions: including the actions of oxytocin that is presumably still secreted from the posterior pituitary in pulses following the continuing burst‐firing of oxytocin neurones during suckling. Notably, this continued burst‐firing of oxytocin neurones during suckling with peripheral actions of oxytocin blocked by F792 reinforces the long‐standing inference that the effectiveness of the suckling stimulus in evoking burst‐firing is a result of the sensory neural input activated by suckling and not a consequence of a systemic action of oxytocin. For example, Wakerley and Lincoln 4 found that i.v. injections of oxytocin did not influence ongoing burst‐firing of oxytocin neurones during suckling.

A different prediction follows from the present study with regard to the secretion and action of oxytocin in parturition. F792 has a significantly improved potency, duration of action and bioavailability 34, 35 compared to atosiban, which has had limited success in delaying pre‐term birth in women experiencing pre‐term labour 23, 24, 25, 26. As indicated above, there is substantial evidence available from human and animal studies indicating an important role in parturition of oxytocin, including its secretion in a pulsatile manner 2, 19. Similar to the pulsatile secretion of oxytocin in response to suckling in lactation, pulsatile oxytocin secretion during birth has been recorded in rats, and was shown to be a consequence of burst firing of magnocellular oxytocin neurones 27, and this evidently involves local auto‐excitatory oxytocin actions because birth is slowed by an oxytocin antagonist given into the magnocellular nuclei 28. However, this activity of the neurones and the subsequent secretion of an oxytocin pulse is associated with a birth, indicating that, during parturition, oxytocin neurones are stimulated by positive‐feedback neural signals from the birth canal, which is distended as a foetus is born 38. Hence, if births are interrupted by the action of an oxytocin antagonist on the myometrium, the positive‐feedback signal will be lost and oxytocin neurone activity will subside. It follows that, in contrast to the expected continuation of secretion of oxytocin pulses during suckling in lactation with blockade of peripheral actions by an oxytocin antagonist, in late pregnancy (e.g. pre‐term labour), the antagonist will effectively, but indirectly, inhibit oxytocin secretion and conserve the posterior pituitary store of oxytocin. This remains to be tested.

The key conclusion from the present study is that F792, when given peripherally in a dose supramaximal for blocking peripheral oxytocin actions, does not enter the brain. Consequently, oxytocin‐dependent processes in the brain in late pregnancy, when a peptide oxytocin antagonist is likely to be used clinically, are unlikely to be affected by peripheral antagonist administration. This should be a primary concern in the design and testing of any oxytocin antagonist, regardless of whether it comprises a peptide or especially a nonpeptide that is more likely to be able to enter the brain 39. There are several actions of oxytocin within the brain in pregnancy and postpartum that are important for a successful outcome of pregnancy. These include an important role for oxytocin released in late pregnancy within the magnocellular nuclei with respect to inducing, along with the actions of oestrogen, changes in oxytocin neurone electrical properties and the local circuitry regulating oxytocin neurones, which are both important for their co‐ordinated burst‐firing during parturition and lactation 2, 40. Clearly, an oxytocin antagonist that enters the brain may interfere adversely with these preparations and impair pulsatile oxytocin secretion in parturition and milk transfer to the newborn. Oxytocin released in the brain during birth has important actions for ensuring the rapid expression of maternal behaviour after birth 3, 33. Furthermore, oxytocin in the brain has more general actions in facilitating social affiliation 3, 41, 42 and any negative action of an oxytocin antagonist on this process, especially around birth, would clearly be an unwanted side‐effect.

Investigations of the multiple roles in behaviours and abnormal mental states that have been postulated for oxytocin have gained popularity recently. Many of these studies have involved insufflating large doses of oxytocin intranasally, in the expectation that the peptide will reach the brain, by an undefined direct route 43, 44, 45. There are transport mechanisms for some peptides that enable transport across the blood–brain barrier 46, although none has been identified for oxytocin, and an action of oxytocin at circumventricular organs has not been described. Even after raising circulating concentrations of oxytocin massively, the integrity of the blood–brain barrier is intact 47 and there is negligible transfer from blood to CSF 48, 49 (at least under normal conditions). It is, however, possible that oxytocin given intranasally (or by any other systemic route) will have peripheral actions, leading indirectly to central effects 45. To exclude such peripheral actions, it would be appropriate to make use of an oxytocin antagonist that potently blocks peripheral actions but which does not enter the brain in studies with intranasal oxytocin.

In principle, an appropriate way of investigating the actions of oxytocin in the brain, apart from the use of genetically modified animals, would be to study the effects of an oxytocin antagonist. Many peptide oxytocin antagonists have been synthesised 25, 26 and, as in the present study, were shown to be active *in vivo*, including in the brain in animal models 50, and against peripheral oxytocin actions. To our knowledge, none of these peptide antagonists have been shown to enter the brain, and so they are not useable for studies of oxytocin actions in the brain in humans. From the present study, F792 also is not suitable for exploring oxytocin actions in the brain in humans: indeed, should peripheral administration of this peptide antagonist, with or without prior oxytocin administration, produce effects on brain function, these are expected to be a result of antagonism of peripheral, not central, actions of oxytocin. Hence, the present study demonstrates that F792 has properties appropriate for use as a selective antagonist of peripheral oxytocin actions without entering the brain and compromising the activity of oxytocin systems therein.
